# The complete genome sequence of an alphabaculovirus from *Spodoptera exempta*, an agricultural pest of major economic significance in Africa

**DOI:** 10.1371/journal.pone.0209937

**Published:** 2019-02-08

**Authors:** Shannon R. Escasa, Robert L. Harrison, Joseph D. Mowery, Gary R. Bauchan, Jenny S. Cory

**Affiliations:** 1 Laboratory for Molecular Ecology, Great Lakes Forestry Centre and Biology Department, Algoma University, Sault Ste, Marie, Ontario, Canada; 2 Invasive Insect Biocontrol and Behavior Laboratory, Beltsville Agricultural Research Center, USDA Agricultural Research Service, Beltsville, Maryland, United States of America; 3 Electron and Confocal Microscopy Unit, Beltsville Agricultural Research Center, USDA Agricultural Research Service, Beltsville, Maryland, United States of America; 4 Department of Biological Sciences, Simon Fraser University, University Boulevard, Burnaby, British Columbia, Canada; Jamia Hamdard, INDIA

## Abstract

Spodoptera exempta nucleopolyhedrovirus (SpexNPV) is a viral pathogen of the African armyworm, *Spodoptera exempta* (Lepidoptera: Noctuidae), a significant agricultural pest of cereal crops in Africa. SpexNPV has been evaluated as a potential insecticide for control of this pest and has served as the subject of research on baculovirus pathology and transmission. Occlusion bodies (OBs) of SpexNPV isolate 244.1 were examined, and the nucleotide sequence of the genome was determined and characterized. SpexNPV-244.1 OBs consisted of irregular polyhedra with a size and appearance typical for alphabaculoviruses. Virions within the polyhedra contained 1–8 nucleocapsids per unit envelope. The SpexNPV-244.1 genome was comprised of a 129,528 bp circular sequence, in which 139 ORFs were annotated. Five homologous regions (*hr*s) consisting of a variable number of 28-bp imperfect palindromes were identified in the genome. The genome sequence contained the 38 core genes of family *Baculoviridae*, as well as three ORFs unique to the SpexNPV sequence and one ORF that was apparently acquired by horizontal gene transfer with a betabaculovirus ancestor. Phylogenetic inference with core gene amino acid sequence alignments placed SpexNPV-244.1 in a lineage containing alphabaculoviruses of *Spodoptera frugiperda* and *Spodopotera exigua* which in turn is part of a larger group of alphabaculoviruses from the subfamily Noctuinae in the lepidopteran family Noctuidae. Kimura-2-parameter pairwise nucleotide distances indicated that SpexNPV-244.1 represented a different and previously unlisted species in the genus *Alphabaculovirus*. Gene parity plots indicated that the gene order of SpexNPV-244.l was extensively collinear with that of Spodoptera exigua NPV (SeMNPV). These plots also revealed a group of 17 core genes whose order was conserved in other alpha- and betabaculoviruses.

## Introduction

The African armyworm, *Spodoptera exempta*, occurs throughout sub-Saharan Africa, Asia, Australia, and New Zealand. It is a major pest in southern and eastern Africa, where it feeds on a variety of cultivated and pasture species of grasses and cereals [1]. Larvae of the African armyworm undergo density-dependent polyphenism, a phenomenon in which population density affects behavior, morphology, and physiology [2]. *S*. *exempta* larvae can occur in either a light-colored “solitary” phase in low-density populations or a darker, more mobile, “gregarious” phase in high-density populations. During outbreaks of *S*. *exempta*, the gregarious phase occurs in huge numbers that migrate from field to field and can cause major damage to pastures and crops. After outbreaks, populations of *S*. *exempta* persist in the low-density solitary phase [1].

A baculovirus that causes disease and mortality in *S*. *exempta* larvae has been identified [3]. Baculoviruses are insect-specific pathogens classified in *Baculoviridae*, a family of viruses with rod-shaped virions and large DNA genomes [4, 5]. Two genera of this family, *Alphabaculovirus* and *Betabaculovirus*, contain lepidopteran-specific viruses that have been developed as environmentally safe biopesticides due to their role as naturally occurring mortality factors for a number of significant lepidopteran pest species [6, 7]. Studies on the African armyworm baculovirus, Spodoptera exempta nucleopolyhedrovirus (SpexNPV), indicate that it is an alphabaculovirus with the potential to serve as an alternative to chemical insecticides for controlling *S*. *exempta* outbreaks [8–9]. Field trials of SpexNPV involving aerial and ground applications in pastures at rates typically used for other baculoviruses yielded consistent control of larval popoulations [10].

SpexNPV has been a frequent subject of research into baculovirus pathology and host susceptibility, particularly in relation to the two different phases of *S*. *exempta* larvae. Gregarious larvae exhibit both a greater resistance to infection by SpexNPV [11] and a lower transmission parameter in the field compared to solitary larvae [12]. Covert, vertically-transmitted infection of larvae by SpexNPV has been observed [13], and vertical transmission was higher from adult moths of solitary larvae that had survived infection with SpexNPV compared to adults of surviving gregarious larvae [14]. Increased susceptibility to SpexNPV infection and viral mortality among *S*. *exempta* larvae was also found to be associated with infections of larvae by *Wolbachia* bacterial symbionts [15]. Genotypic and phenotypic variability within populations of SpexNPV has been documented [16–17]. However, very little nucleotide sequence data have been reported for SpexNPV.

In this study, we report the determination and characterization of the genome sequence of an isolate of SpexNPV. We describe the properties of the SpexNPV OBs and genome, and the relationships of SpexNPV to other alphabaculoviruses.

## Materials and methods

### Virus extraction

SpexMNPV occlusion bodies (OBs) were obtained from a bottle of virus-killed *S*. *exempta* cadavers labeled SpexMNPV 244.1 01/01/05. The original virus isolate was collected in Tanzania in the early 1970’s, amplified in *S*. *exempta* larvae and stored at -20°C. This stored isolate was re-amplified in *S*. *exempta* to produce the material used for electron microscopy and DNA sequencing. Approximately 30 ml of larval material were placed in a beaker and 10% SDS was added to a final concentration of 0.3% SDS and the mixture stirred for 2 h. Large debris was filtered through cheesecloth and the crude OB suspension was centrifuged for 30 min/2500 rpm/17°C (rotor JS 5.3). The pellet was washed and re-spun. After the 3rd wash, the OBs were re-suspended in water and loaded onto 45% sucrose cushions and centrifuged at 8000rpm/30min/15°C. The pellet was re-suspended in water and placed on a 60 /50 /30% discontinuous sucrose gradient and ultra-centrifuged at 150000 rpm/2 h/15°C. The OB band was collected, diluted with water and centrifuged for one h at 4000 rpm/ 15°C. The supernatant was removed and the pellet was re-suspended in 5 ml of water and counted using a haemocytometer. The final OB count was 1.925 X 10^10^ OBs/ml. This isolate was named SpexNPV-244.1.

### Electron microscopy

SpexNPV-244.1 OBs were subjected to cryofixation in a Quorum PP2000 cryo-prep chamber (Quorum Technologies, East Sussex, UK) and visualized with an S-4700 field emission scanning electron microscope (Hitachi High Technologies America, Inc., Dallas, TX, USA) as previously described [18]. For transmission electron microscopy, OBs were subjected to chemical fixation and embedded in LX-112 resin as previously described [18]. Ultrathin sections of embedded OBs were cut and visualized with a Hitachi HT-7700 transmission electron microscope (Hitachi High Technologies America, Inc., Dallas, TX, USA).

### Virus DNA isolation and sequencing

DNA extraction was performed using 3 ml of the purified OB mixture. The volume was increased to 10 ml by the addition of 4M sodium thioglycollate and 1M sodium carbonate plus water. The suspension was centrifuged briefly to remove small debris and loaded onto a continuous 45/10% sucrose gradient. The gradients were ultra-centrifuged at 20000 rpm/1.25 h/4°C and the virion band was extracted by syringe. The virions were diluted with 1 X TE and ultra-centrifuged at 22000 rpm/2 h/4°C. A blue pellet was visible and was re-suspended in TE; 25 μl of proteinase K (20 mg ml ^-1^) was added and the suspension was incubated at 37°C for 30 min. Another 25 μl of proteinase K was added along with 10% SDS to a final concentration of 1% SDS and the mix incubated at 50°C. The solution was phenol/chloroform-extracted followed by dialysis in changes of 2 L of 1 X TE for 36 h. Gel electrophoresis and concentration readings gave a final value of 151 ng ul ^-1^.

A total of 20 μg of DNA was sent for sequencing (Greenomics/Applied Bioinformatics, Wageningen, The Netherlands). A shotgun library of SpexNPV viral DNA was constructed. Clones from this library were sequenced in both directions until a genomic coverage of approximately 8 times was reached requiring approximately 1440 total reads. The average length of the sequence reads was 750 nt. All sequences were assembled into contigs, and these contigs were edited to an error level of less than 1 error per 10,000 nucleotides. Gaps were closed by primer walking.

### ORF and homologous region (*hr*) annotation

Sequence analysis was carried out by using NCBI open reading frame finder (ORF Finder) [19], BLAST [20] and Lasergene GeneQuest (DNASTAR). ORFs were considered for further analysis if they encoded 50 amino acids or more and were initiated with a methionine codon. Repeated sequences of putative homologous regions (*hr*s) were identified with Tandem Repeats Finder [21] and Reputer [22]. Promoter regions were found with the aid of Promoter Scan [23].

### Sequence comparison and phylogeny

Gene parity plot analysis [24] was done to compare the gene order between SpexNPV-244.1 and Autographa californica multiple nucleopolyhedrovirus C6 (AcMNPV-C6) [25], Spodoptera litura nucleopolyhedrovirus G2 (SpltNPV-G2) [26], Spodoptera exigua multiple nucleopolyhedrovirius US1 (SeMNPV-US1) [27], and Cydia pomonella granulovirus M1 (CpGV-M1) [28].

For phylogenetic inference based on core gene sequences, the conceptual amino acid sequences of the 38 core genes found in SpexNPV-244.1 and other baculovirus genomes (S1 Table) were aligned using MUSCLE [29] as implemented in LaserGene MegAlign Pro 15 (DNASTAR) with default parameters. The amino acid alignments were concatenated using BioEdit 7.1.3 [30]. Phylogenetic trees were inferred by both the minimum evolution (ME) method using MEGA7 [31] with the JTT substitution matrix and a gamma distribution shape parameter of 0.8398, and the maximum likelihood (ML) method with RAxML [32] using the LG model. The accuracy of the trees was evaluated by 500 bootstrap replicates for the ME tree and 100 bootstrap replicates for the ML tree.

Phylogenetic analysis of SpexNPV-244.1 ORF91 and other homologs of this ORF was carried out as described above, except the gamma shape parameter for ME analysis was 1.4762, and the ML analysis was performed with MEGA7 using the JTT substitution model.

Phylogenetic analysis also was carried out with the COI-5P region of mitochondrial DNA (i.e. the 5′ end of the cytochrome c oxidase subunit I gene; [33]) of baculovirus hosts. Sequences were aligned with MUSCLE, and phylogenetic inference was performed as for the ORF91 alignment described above, with TN93 substitution model used for ME phylogeny with a gamma shape parameter value of 0.5566 and the GTR model used for ML phylogeny.

Pairwise Kimura-2-parameter nucleotide distances between the partial nucleotide sequences of the lef-8, lef-9, and polh genes of SpexNPV-244.1 and other alphabaculoviruses were determined as previously described [34] using MEGA7.

## Results

### SpexNPV-244.1 occlusion bodies contain multicapsid virions

**Fig 1:**
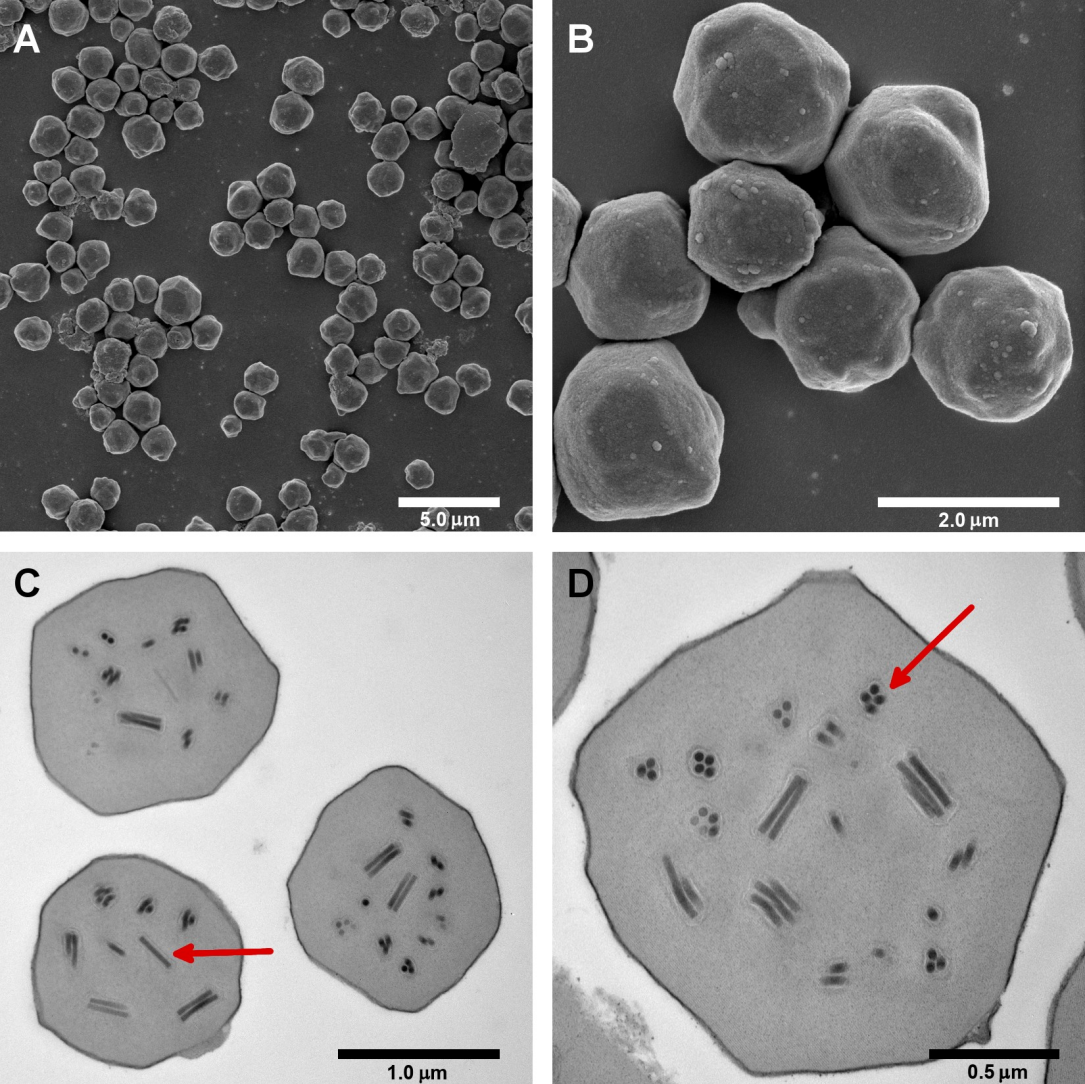
Occlusion bodies (OBs) of baculovirus isolate SpexNPV-244.1. (A, B) Scanning electron micrographs of SpexNPV-244.1 OBs. (C, D) Transmission electron micrographs of ultrathin sections through SpexNPV-244.1 OBs. The red arrow in (C) indicates a longitudinal section through a virion containing a single nucleocapsid, while the red arrow in (D) indicates a cross-section through a virion containing four nucleocapsids. The lengths of scale bars are given beneath each scale bar.

OBs of the SpexNPV-244.1 isolate exhibited an irregular polyhedral shape typical of alphabaculovirus OBs (Fig 1A, Fig 1B). OBs in SEM images measured from 1.3 to 1.9 μm in diameter. The OBs contained multiple virions, with each virion containing between one and eight nucleocapsids (Fig 1C, Fig 1D). The multicapsid nature of the occluded virions also was observed in a previously published micrograph of an *S*. *exempta* baculovirus [8]. The nucleocapsids in TEM cross sections measured approximately 33 nm x 258 nm.

### The SpexNPV-244.1 genome contains an unusually long *ie-1* ORF

The SpexNPV genome sequencing reads were assembled into a final contig of 129,528 bp, with a nucleotide distribution of 41.23% G+C. One hundred thirty-nine ORFs were annotated in the genome sequence. The SpexNPV-244.1 is smaller than other representative *Spodoptera* spp. alphabaculoviruses, and less GC-rich than all except Spodoptera frugiperda multiple nucleopolyhedrovirus 3AP2 (SfMNPV-3AP2; Table 1).

**Table 1:** Characteristics of genomes of *Spodoptera* spp. Alphabaculoviruses.

Virus name	Genome size, bp	% G+C	Number of annotated ORFs	Number of *hr*s	Reference
Spodoptera exigua multiple nucleopolyhedrovirus US1^a^	135611	43.81	139	5	[27]
Spodoptera litura nucleopolyhedrovirus G2^a^	139342	42.77	141	17	[26]
Spodoptera frugiperda multiple nucleopolyhedrovirus 3AP2^a^	131331	40.24	143	8	[35]
Spodoptera littoralis nucleopolyhedrovirus AN1956^a^	137998	44.67	132	15	[36]
Spodoptera litura nucleopolyhedrovirus II	148634	45.0	149	7	Unpublished (accession no. EU780426)
Spodoptera exempta nucleopolyhedrovirus 244.1	129528	41.23	139	5	This study (accession no. MH717816)

^a^Representative isolates of alphabaculovirus species currently recognized by the ICTV.

**Fig 2:**
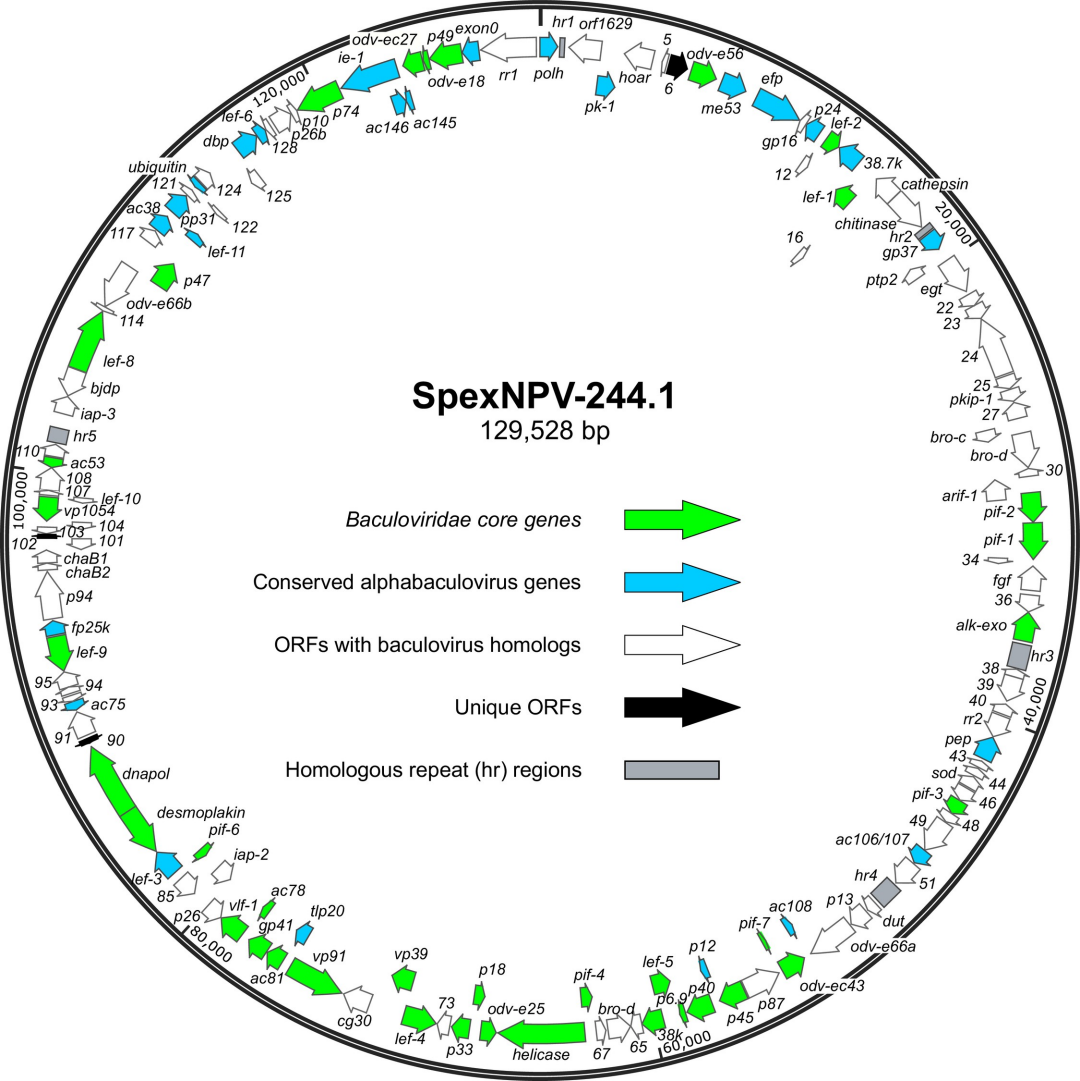
Map of the ORFs and other features of the SpexNPV-244.1 genome. ORFs are represented by arrows that indicate the orientation the ORF relative to the *polyhedrin* (*polh*) gene. The identity of each ORF is designated by a name (for conserved or well-characterized baculovirus homologs) or the number given to the ORF indicating its order in the annotation. A color key indicates different categories of ORFs in the figure, as well as homologous regions (*hr*s).

With respect to the *polyhedrin* (*polh*) ORF, there were 75 ORFs in the clockwise (*polh*-sense) direction and 64 in the counterclockwise direction (S2 Table, Fig 2). The ORFs include the 38 core genes found in all baculovirus genomes to date [37, 38]. At first examination, it appeared that SpexNPV-244.1 did not contain a homolog of the *ac146* ORF, which has been previously identified in all lepidopteran baculoviruses but not in the dipteran or hymenopteran baculoviruses [37]. However, when examining its location in other lepidopteran baculovirus genomes, it was consistently located adjacent to the *ie-1* gene. In the Choristoneura occidentalis granulovirus genome, the *ac146* and *ie-1* sequences overlap each other [39]. Upon further investigation of the SpexNPV-244.1 *ie-1* sequence, a homolog to *ac146* was found entirely within the *ie-1* gene but in the opposite orientation. The *ie-1* gene (ORF132) extended from 120,978<123,473 nt, while the *ac146* homologue was located from 122,839>123,429 nt. ORF132 encodes a predicted 831-amino acid gene product, which is significantly longer than the 714-amino acid IE-1 protein encoded by SeMNPV-US1 or the 682-amino acid IE-1 protein encoded by SfMNPV-3AP2. The extra sequence in the SpexNPV-244.1 IE-1 product occurs at the N-terminus, suggesting that the unusual size of the SpexNPV-244.1 *ie-1* ORF is due to an upstream ATG codon that is in-frame with the *ie-1*-homologous coding sequence of ORF132. No early promoter motif was identified for ORF132, which suggests that transcription and translation of the SpexNPV-244.1 *ie-1* gene initiate downstream of the ORF132 start codon. The other 24 ORFs reported by Garavaglia et al. [37] to be in all alphabaculovirus genomes were identified in the SpexNPV-244.1 sequence.

Most of the ORFs in the SpexNPV-244.1 genome were represented by homologs in other baculovirus genomes. Three ORFs–ORF6, ORF90, and ORF102 –appeared to be unique. BLASTp and HHpred queries with the predicted amino acid sequences of these ORFs failed to yield convincing evidence of significant sequence identity with other sequences in databases, or of the presence of characterized domains. ORF6 is 264 codons long (excluding the stop codon) and is preceded by early and late promoter motifs (S2 Table). ORFs 90 and 102 are shorter (74 and 53 codons, respectively). ORF90 is preceded by an early promoter motif, while no promoter motifs are associated with ORF102.

**Fig 3:**
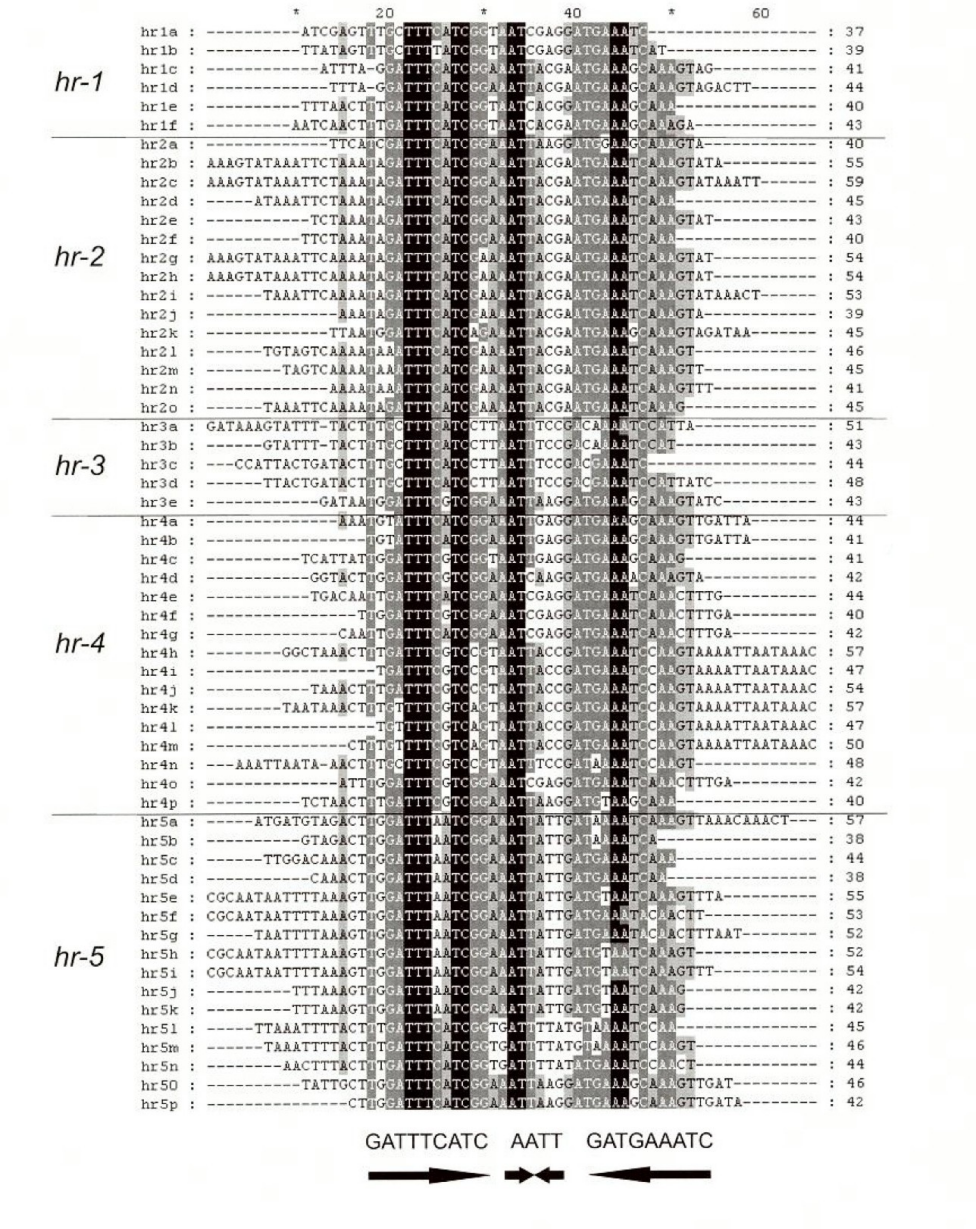
SpexNPV-244.1 homologous region (*hr*) unit repeats. An alignment of the individual repeats of the 5 SpexNPV-244.1 *hr*s are shown. The consensus sequence is located below the alignment. Shading corresponds to the degree of nucleotide conservation: black, 100%, dark grey, >85%, light grey, > 60%.

Five homologous regions (*hr*s; [40]) were identified in the SpexMNPV sequence (S2 Table, Fig 2). The SpexNPV *hr*s were dispersed throughout the genome and consisted of five to sixteen repeats of an imperfect 28 bp palindromic sequence (Fig 2, Fig 3). The SpexNPV palindrome consensus sequence resembled the consensus sequences of *hr* repeats from other related alphabaculoviruses [18], and exhibited 57.6% sequence identity with the *hr* consensus sequence of SeMNPV-US1. The positions of *hr*s 3, 4, and 5 in the SpexNPV-244.1 genome were conserved with the positions of *hr*s 2, 3, and 5 of the SeMNPV-US1 genome relative to homologs present in both genomes. SpexNPV *hr2* was located in a position between the *chitinase* and *gp37* genes that is conserved with *hr2* of the SfMNPV-3AP2 genome [35]. This location was found to be the site of homologous recombination between SfMNPV isolate ColA and an ancestor of virus isolate Spodoptera litura nucleopolyhedrovirus II (SpltNPV-II; [41]).

### SpexNPV-244.1 is most closely related to *S*. *frugiperda* and *S*. *exigua* alphabaculoviruses

**Fig 4:**
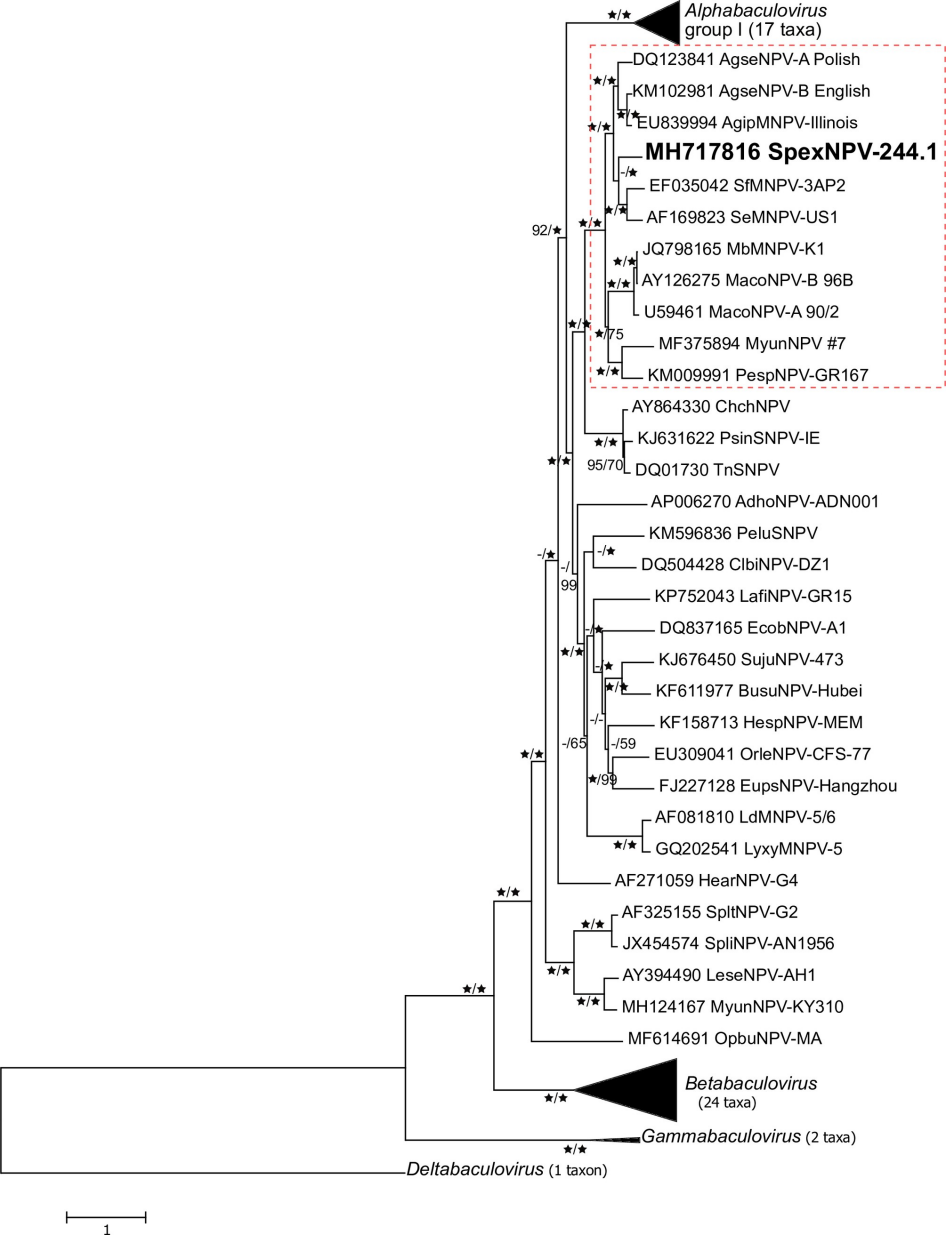
Phylogenetic inference of the relationships among SpexNPV-244.1 and representative isolates of other baculovirus species. Concatenated alignments of 38 baculovirus core gene amino acid sequences were used to construct ML and ME trees as described under **Materials and Methods**. Shown is the ML tree with bootstrap values ≥50% for branches in trees produced by both ME and ML methods (displayed as ME/ML). Stars indicate bootstrap values of 100%. Branches for the group I viruses of genus *Alphabaculovirus* and for the viruses of genera *Betabaculovirus* and *Gammabaculovirus* are collapsed. A red box with dashed lines indicates the cluster of viruses from subfamily Noctuinae (Lepidoptera: Noctuidae) into which SpexNPV-244.1 was placed.

The top matches of BLASTx queries with the ORFs of SpexNPV-244.1 were with ORFs from alphabaculovirus isolates from host species of the genera *Spodoptera* and *Agrotis*, suggesting that SpexNPV is closely related to these viruses. Phylogenetic inference based on concatenated core gene amino acid sequence alignments placed SpexNPV-244.1 in a clade with SeMNPV-US1 and SfMNPV-3AP2 (Fig 4). This clade of *Spodoptera* spp. alphabaculoviruses was in turn part of a larger clade that also included *Agrotis* spp. alphabaculoviruses. The *Agrotis*/*Spodoptera* clade is part of a larger group consisting of alphabaculoviruses from the subfamily Noctuinae of lepidopteran family Noctuidae [18]. This group of viruses from species of Noctuinae represents approximately one-third of the group II alphabaculovirus taxa that have been sequenced.

Pairwise Kimura-2-parameter distances for partial *lef-8*, *lef-9*, and *granulin* nucleotide sequences between SpexNPV-244.1 and the representative isolates of currently recognized alphabaculovirus species were estimated to determine if SpexNPV-244.1 represented a new species. Pairwise distances between SpexNPV-244.1 and other viruses in the *Agrotis*-*Spodoptera* virus clade ranged from 0.244–0.332 substitutions/site for *polh*, 0.645–0.776 substitutions/site for *lef-8*, and 0.242–0.313 substitutions/site for *lef-9*. The nucleotide distances were larger in pairwise comparisons of SpexNPV-244.1 sequences with other alphabaculoviruses. The estimated distances exceed the 0.050 substitutions/site demarcation limit proposed for separating two baculovirus species [42], indicating that SpexNPV-244.1 likely represents a previously undescribed species of genus *Alphabaculovirus*.

Partial *lef-8* and *lef-9* sequences reported by Thézé et al. [43] for SpexNPV isolate k11 were almost completely identical to the corresponding sequences in SpexNPV-244.1, with only a single nucleotide mismatch distinguishing the isolates at these two loci. The SpexNPV-k11 isolate is part of the reference virus collection at the Centre for Ecology and Hydrology (NERC-CEH), Wallingford, UK, and may also originate from Tanzania. Likewise, a 313-bp sequence derived from the *polh* gene of another Tanzanian isolate, SpexNPV-var1 [44], was identical to the corresponding region of the SpexNPV-244.1 *polh* gene. However, the partial *polh* sequence for SpexNPV-k11 differed from the corresponding sequence in SpexNPV-244.1 by mismatches at seven positions, with six of those mismatches occurring within the first 20 bp of the alignment. The SpexNPV-k11 *polh* sequence encodes a valine at codon 61 of the gene, while SpexNPV-244.1 and related viruses (SeMNPV-US1, SfMNPV-3AP2, AgseNPV-A, and AgipMNPV) encode a leucine at this position, which raises the possibility that the SpexNPV-k11 sequence contains errors at one end of the sequence file.

**Fig 5:**
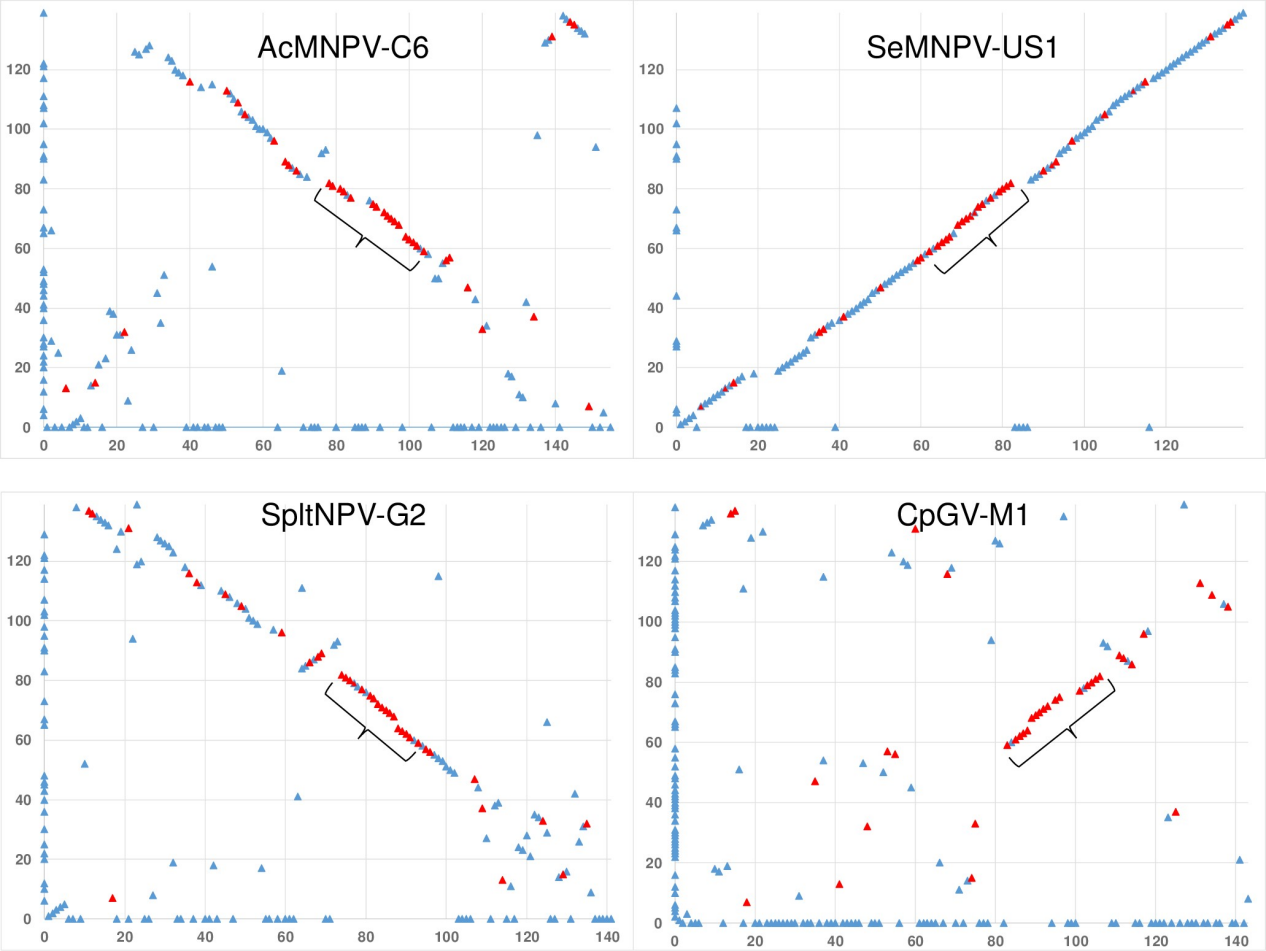
Gene parity plots comparing the ORF content and order of SpexNPV-244.1 and representative alpha- and betabaculoviruses. Plots compare the ORF content and order of the SpexNPV-244.1 genome (y-axis) with that of AcMNPV-C6, SeMNPV-US1, SpltNPV-G2, and Cydia pomonella granulovirus M1 (CpGV-M1) (x-axes). Each point in the plot represents an ORF, and points corresponding to ORFs present in only one of the compared genomes appear on the axis for the virus in which they are present. ORFs corresponding to core genes of family *Baculoviridae* are represented by red triangles, and other ORFs are represented by blue triangles. A cluster of core genes with an order conserved in all five viruses are indicated with a bracket.

Gene parity plot analysis revealed an extensive degree of collinearity between the ORFs of SpexNPV-244.1 and SeMNPV-US1 (Fig 5). The SpexNPV x SeMNPV plot illuminated the absence of SeMNPV ORFs 20 to 24 and 83 to 86 in the SpexNPV-244.1 genome, which may explain the relatively small size of the SpexNPV-244.1 genome (Table 1). SpexNPV-244.1 *hr2* occupies the location of SeMNPV ORFs 20 to 24, suggesting that a recombination event involving this *hr* resulted in the acquisition of these ORFs in an ancestor of SeMNPV. The region of the SeMNPV-US1 genome extending from ORFs 83 to 86 includes a putative non-*hr* origin of replication that is characterized by multiple short palindromes and direct repeats [45]. It is possible that a recombination event involving this potential origin resulted in the loss of the sequence encompassing ORFs 83 to 86 in an ancestor of SpexNPV.

**Fig 6:**
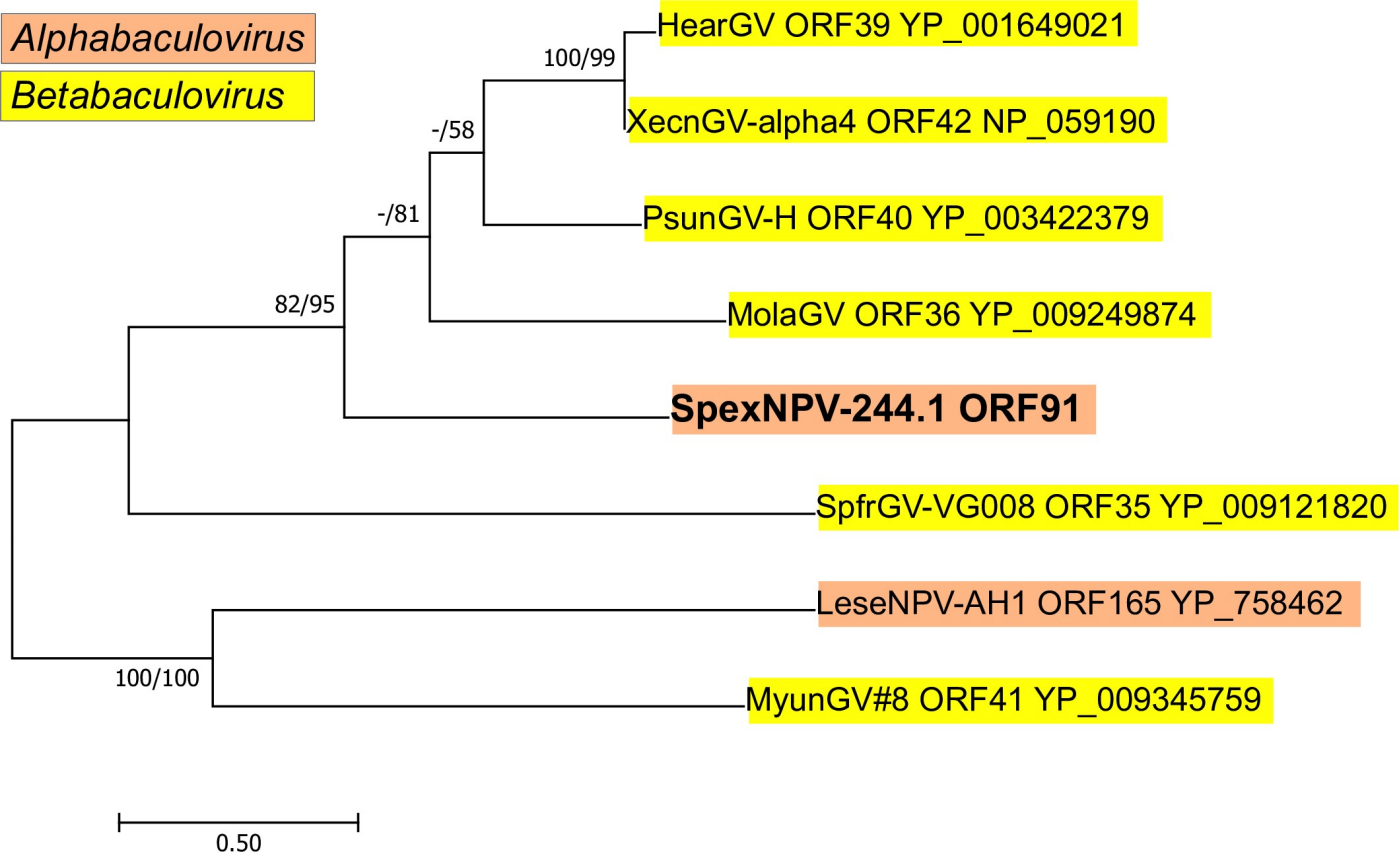
Phylogenetic analysis of SpexNPV-244.1 ORF91 homologs. An ML phylogram inferred from a MUSCLE alignment of amino acid sequences encoded by homologs of SpexNPV-244.1 ORF91 is shown. GenBank accession numbers for the homologs are listed next to the taxon abbreviations. Bootstrap values (≥50%) are indicated at interior branches for ME and ML analysis (presented as ME/ML) where they occur, with a dash (-) indicating when the branch was not supported in either the ME or ML tree. The baculovirus genus of each taxon in the tree is indicated with a color-coded text background.

No homologs for SpexNPV-244.1 ORF91 were detected in any of the four viruses that were compared by gene parity plot analysis. BLASTx queries with this ORF only yielded matches with an ORF from Leucania separata nucleopolyhedrovirus AH1 (LeseNPV-AH1) and some betabaculovirus genomes. A query with HHpred did not produce convincing evidence for any characterized domains in the ORF91 amino acid sequence. Phylogenetic inference with homologous amino acid sequences placed ORF91 in a group with sequences from betabaculovirus clade *a* [46] (Fig 6). A prior analysis of the ORF91 homolog in the genome of Mythimna unipuncta granulovirus #8 (MyunGV#8) suggested that the LeseNPV-AH1 homolog of this ORF may have been acquired from an ancestor of MyunGV#8 [47]. ORF91 was not grouped with the MyunGV#8 and LeseNPV-AH1 homologs, suggesting that SpexNPV-244.1 may have acquired ORF91 from an ancestral clade *a* betabaculovirus during an independent horizontal gene transfer event.

A noticeably lower degree of collinearity was evident in the gene parity plots with AcMNPV-C6, SpltNPV-G2, and the betabaculovirus isolate CpGV-M1 (Fig 5). These three plots (as well as the plot with SeMNPV-US1) revealed that seventeen of the 38 core genes of *Baculoviridae* were arranged in an order that was conserved in all five of the virus genomes analyzed in this study. This group of genes includes the four-gene cluster of *ac95* (*helicase*), *ac96* (*pif-4*), *ac98* (*38k*), and *ac99* (*lef-5*), whose order had been previously reported to be conserved in a wide range of baculovirus genomes, including the genome of the deltabaculovirus Culex nigripalpus nucleopolyhedrovirus (CuniNPV) [48]. In addition to these four core genes, the larger cluster of core genes with a conserved order also contained *ac77* (*vlf-1*), *ac78*, *ac80* (*gp41*), *ac81*, *ac83* (*vp91*), *ac89* (*vp39*), *ac90* (*lef-4*), *ac92* (*p33*), *ac93* (*p18*), *ac94* (*odv-e25*), *ac100* (*p6*.*9*), *ac101* (*p40*), and *ac103* (*p48*). Genes of this cluster were previously revealed by gene parity plot analysis of the Catopsilia pomona nucleopolyhedrovirus to possess a conserved order among lepidopteran baculoviruses [49]. The order of the core genes in this cluster was not conserved in gammabaculoviruses or in CuniNPV [50].

## Discussion

Early restriction endonuclease studies on a SpexNPV isolate from diseased *S*. *exempta* larvae collected in Kenya had led the authors of those studies to conclude that SpexNPV was a genomic variant of AcMNPV [51, 52]. However, an analysis of the complete genome sequence of an isolate of SpexNPV, reported for the first time in this study, has shown unambiguously that an alphabaculovirus distinct from AcMNPV infects African armyworm larvae. AcMNPV variants previously have been reported from a wide range of lepidopteran host species [53, 54], but partial sequences of other SpexNPV isolates match the sequence of the SpexNPV-244.1 isolate reported here [43, 44], suggesting that SpexNPV-244.1 is more representative of the baculovirus pathogens found in populations of *S*. *exempta*.

**Fig 7:**
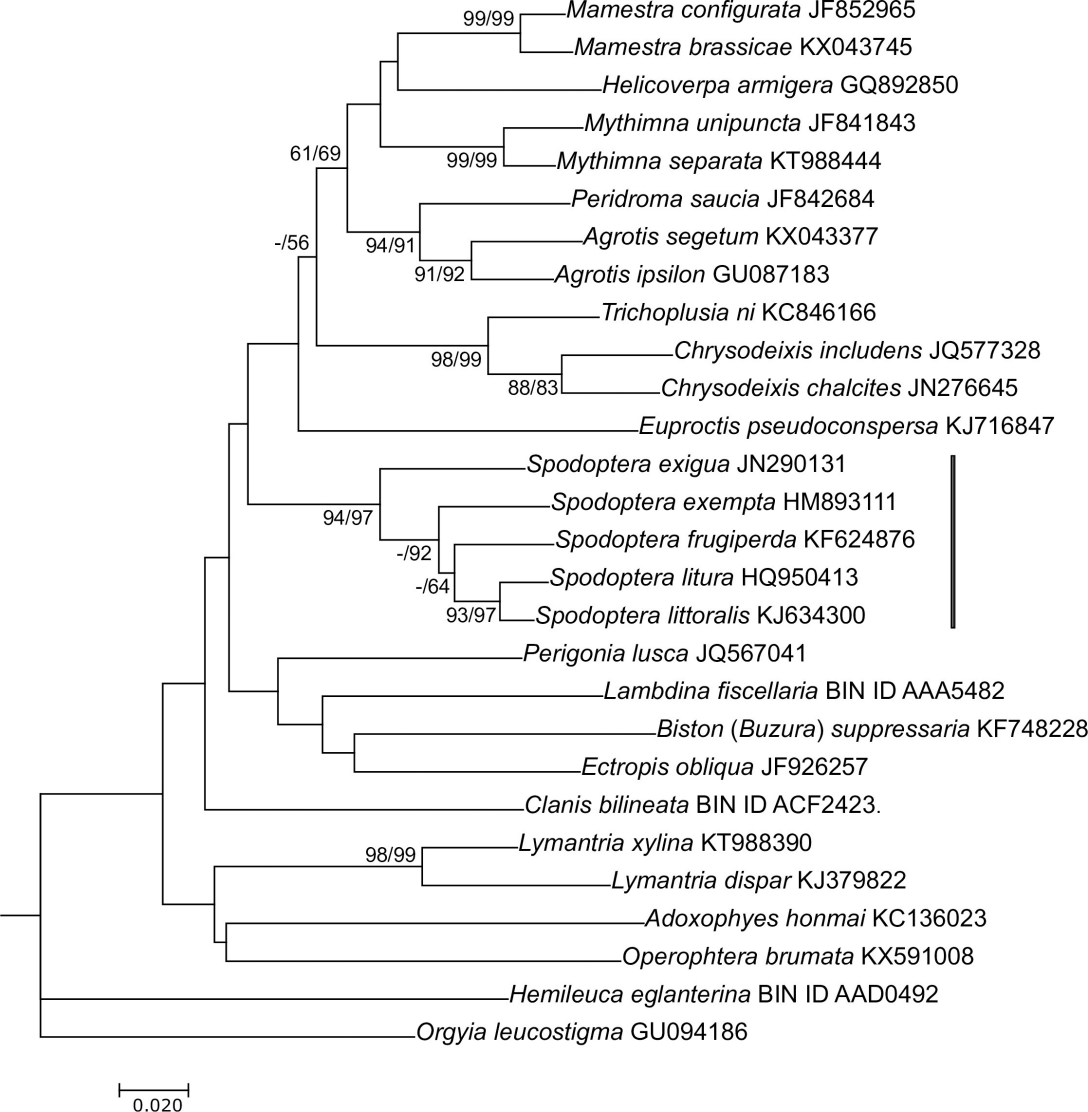
Phylogenetic analysis of mitochondrial sequences from group II alphabaculovirus host species. An ML phylogram inferred from a MUSCLE alignment of mitochondrial nucleotide sequences from the 5′ end of the mitochondrial gene cytochrome c oxidase subunit I (COI-5P; [33]) is shown. Either GenBank abbreviation numbers or Barcode of Life Database BIN ID numbers are listed next to the species names. Bootstrap values (≥50%) are indicated as described for Fig 6. A vertical line delineates the clade of *Spodoptera* species. A COI-5P sequence from *Apis mellifera* (GenBank accession no. KR895607) was used as an outgroup.

Alphabaculoviruses from host *Spodoptera* spp. occur in two lineages in core gene phylogenetic trees, with SpexNPV-244.1 placed in one lineage with alphabaculoviruses isolated from *S*. *frugiperda* and *S*. *exigua*, and a second lineage containing alphabaculoviruses from *S*. *litura* and *S*. *littoralis* (Fig 7). Phylogenies also place SpltNPV-II in the same lineage as SpexNPV-244.1 [41], but while the genome sequence of SpltNPV-II was deposited in GenBank in 2008, there has been no published description of SpltNPV-II describing this virus and confirming its origin. The distribution of viruses between these two lineages does not reflect the geographic ranges of the host species, as *S*. *exigua*, *S*. *exempta*, *S*. *litura* and *S*. *littoralis* are Old World species while *S*. *frugiperda* is a New World species [55]. Phylogenetic inference of mitochondrial cytochrome oxidase I (COI) nucleotide sequences from the hosts of group II alphabaculoviruses places the five *Spodoptera* hosts in a single clade with *S*. *litura* and *S*. *littoralis* (once considered to be the same species) as sister taxa and *S*. *exigua* as the most basal taxon in the clade (Fig 7). These topological features have also been also observed in other phylogenetic analyses of *Spodoptera* spp. based on morphological characters [55] and molecular data [56]. Comparison of host and virus phylogenetic trees suggests that SpltNPV-G2 and Spodoptera littoralis nucleopolyhedrovirus AN1956 (SpliNPV-AN1956) are part of a narrowly distributed and/or relatively recent lineage, while the lineage containing SpexNPV-244.1, SeMNPV-US1, and SfMNPV-3AP2 is older and/or more widespread among host species of *Spodoptera*.

Although SeMNPV, SfMNPV, and SpexNPV-244.1 are closely related, there are differences in the ORF content of their genomes that may have some role in host range and specificity. Aside from the three ORFs unique to the SpexNPV-244.1 genome, SpexNPV-244.1 contains another 11 ORFs not annotated in SeMNPV-US1, while the SeMNPV-US1 genome contains 14 ORFs not identified in SpexNPV-244.1. SfMNPV-3AP2 contains 21 ORFs not described in SpexNPV-244.1, and SpexNPV-244.1 contains 12 ORFs not found in SfMNPV-3AP2, including 9 also not found in SeMNPV-US1. A homolog of the *lef-7* gene, which encodes an F-box protein that promotes AcMNPV DNA replication [57], is present in both SfMNPV-3AP2 and SeMNPV-US1, but not in SpexNPV-244.1. Other ORFs present only in a subset of the three *Spodoptera* spp. virus genomes include *bro* genes as well as ORFs that are found in other alphabaculoviruses from subfamily Noctuinae (Fig 4).

Differences in non-coding parts of the *Spodoptera* spp. virus genomes may also play a role in specifying host range. The *hr1* sequence of AcMNPV is bound by a 38 kDa host protein, and this binding is required for the enhancer activity of *hr1* in transient expression assays [58]. This finding suggests that host protein interactions may be required for aspects of baculovirus gene expression in a host-specific fashion. However, *hr1* also has been reported to exhibit enhancer activity in mammalian cells [59], which suggests that its enhancer function is not necessarily host-specific.

Determining the contribution of ORFs and non-coding regions to the host range and specificity of *Spodoptera* spp. alphabaculoviruses requires additional experimentation and is further complicated by the results of previously published bioassays showing that *S*. *frugiperda* and *S*. *exigua* larvae exhibit different patterns of susceptibility to *Spodoptera* NPVs [60]. While *S*. *exigua* is susceptible to lethal infection by isolates of both SfMNPV and SeMNPV, *S*. *frugiperda* is refractive to SeMNPV. In addition, there are no published bioassays detailing the pathogenicity of SpexNPV for either *S*. *exigua* or *S*. *frugiperda* larvae, nor of the pathogenicity of SeMNPV and SfMNPV against larvae of *S*. *exempta*.

There is a correlation between the increased cuticular melanization among S. exempta larvae reared in crowded populations to simulate the gregarious phase and increased levels of hemolymph phenoloxidase (PO) activity that may account for the lower susceptibility of crowded S. exempta larvae to SpexNPV infection [11]. Although some studies have found a similar correlation between susceptibility to baculovirus infection and PO levels, [61, 62], other studies have found little or no correlation [63, 64], indicating that PO levels within an insect host are not a reliable indicator of baculovirus susceptibility. SpexNPV isolates are genetically diverse [16], and the different larval phases of *S*. *exempta* may impose unique selection pressures on different genotypes. This selection pressure may involve an interaction with the host immune response, but one that is not necessarily limited to hemolymph PO levels. The effects of selection in the different larval phases on viral genotype composition will require additional experiments to determine.

The data reported in this study provide a reference genome sequence which will serve as the basis for the creation of a new species in genus *Alphabaculovirus* and for further studies into the genetic and phenotypic variability of SpexNPV and its impact on transmission and host susceptibility to viral infection.

## Supporting information

S1 Table Baculovirus genomes used for phylogeny. (XLSX)

S2 Table SpexNPV-244.1 ORFs and hrs. (XLS)
